# Analysis of Tradable Water Volumes of Industry in Water-Rich Areas of China: A Case Study of Changsha City

**DOI:** 10.3390/ijerph18020679

**Published:** 2021-01-14

**Authors:** Xiang-nan Chen, Feng-ping Wu, Fang Li, Yue Zhao, Xia Xu

**Affiliations:** 1 Business School, Hohai University, Nanjing 211100, China; 180213120002@hhu.edu.cn (X.-n.C.); lf@hhu.edu.cn (F.L.); zh_yyyyy@hhu.edu.cn (Y.Z.); 170212070004@hhu.edu.cn (X.X.); 2 Institute of Planning and Decision-Making, Hohai University, Nanjing 211100, China

**Keywords:** water-rights trading, tradable water volumes of industry, tradable control volumes of industry, the subjects of water-rights trading, water-rich areas of China

## Abstract

Tradable water volumes is one of the basic elements of water-rights trading. In China, water-rights transactions mostly occur in water-deficient areas. However, the water-rich areas are also facing serious water-shortage problems. It is necessary to stimulate the water-rights trading power in abundant water areas to improve water-resource predicament. This paper studied the concept and calculation method of tradable water volumes of industry. First, based on the property rights theory, we analyzed the concept of tradable water volumes, and put forward the preliminary determination of water-rights trading subjects. Then, we defined the tradable water volumes of industry as the difference between the initial water-rights allocation and the water demand of industry. We used the proportion method to calculate the initial water-rights allocation of industry under different runoff frequencies, and grey model (1,1) to predict the water demand of industry. Finally, we applied the calculation method to Changsha city which is in a water-rich area of China. The calculation results contribute to water-resource management in Changsha city. This paper will provide a theoretical basis for researching the tradable water volumes, and promote the development of water-rights trading in China’s water-rich areas.

## 1. Introduction

Water resources are not only basic natural resources, but also strategic economic resources. In the past century, human water use has increased six-fold and is still growing at a rate of about 1% per year, water security and climate change will be continuing, and profound crises will face the world in the coming decades [1]. The United Nations 2030 Development Agenda set to “ensure the availability and sustainable management of water and sanitation for all” as the No. 6 Goal of Sustainable Development (SDG) [2]. Obviously, the problem of water resources has drawn worldwide attention. China is also facing a severe water-resource situation. China’s population accounts for 20% of the world’s population, while water resources only account for 7%. There are 16 provinces facing water shortage in China’s 31 provinces and 2/3 of 600 cities are confronted with serious water shortage.

Since the 1980s, the worldwide trend of water marketization is obvious, which has attracted wide attention in academic circles. Howitt [3], Settre and Wheeler [4], Bauer [5] and Easter et al. [6] have studied and summarized the development of water markets in the United States, Australia, Chile, and other countries. Bjornlund [7] summarized the development experience of the water-rights markets in the southwest of the United States, Chile and Victoria of Australia, and put forward suggestions for the construction of water markets in developing countries. Grafton et al. [8] constructed a complete framework of water-rights markets, and identified the strengths and weaknesses of water-rights markets in China, Australia, the United States, South Africa, Chile. Lewis and Zheng [9] analyzed the difficulties in water-rights market reform. China’s water-rights trading started from the transaction between Dongyang city and Yiwu city in Zhejiang province in 2000. Hu and Wang [10] and Shen [11] analyzed the practices of early water-rights trading in China. In 2014, China’s Ministry of Water Resources proposed to carry out different types of water-rights pilot projects in Ningxia, Jiangxi, Hubei, Inner Mongolia, Henan, Gansu, and Guangdong. By the end of 2017, the pilot projects had been basically completed. Liu et al. [12], Wei [13], Hong et al. [14], Tian [15], Zhang [16], and Zhao et al. [17] respectively summarized the practice and experience in the pilot areas of water-rights transaction. Liu et al. [18] and the Development Research Center of the State Council and The World Bank Research Group of “China’s Water Governance” [19] comprehensively summarized the process of domestic water-rights trading, and proposed that the construction of water rights and water markets in China is still at the exploratory stage. In China, water-resource problems in water-deficient areas are more serious than those in water-rich areas [20]. Therefore, the demand for water-rights trading is strong and the water-rights markets have developed rapidly in areas of lacking water. The total amount of water resources is relatively rich, so the impetus for carrying out water-rights trading in water-rich areas is insufficient. With the development of the economy and the change of climate, the areas of sufficient water are also faced with the problems of water shortage [21]. Water-rights trading is one of the most effective means to solve the water-resource dilemma in water-rich areas [22]. Based on the development of water rights and water markets in Jiangxi Province, Zhou et al. [23] analyzed the demand and form of water-rights trading, and provided reference for the development of water-rights markets in abundant water areas. Tian [24] points out that the reform of water-rights system in water-rich areas is still confronted with difficulties, such as weak awareness of water rights, unavailability of scarce water-resource value, low pressure of total water amount control, and imperfect laws and regulations.

The definition of the tradable water volumes (TWV) is one of the basic issues of water-rights trading [25]. According to the analyses of water-rights markets in Brazil, Spain, and Colorado in the United States, Marino and Kemper [26] believed that the establishment of water-rights trading must have sufficient information of the TWV and the infrastructures necessary for measuring and transferring water resources. However, in practice, the determination method of the TWV lacks science. In New South Wales in Australia, only 40% of the water rights in the irrigated areas have been allowed to be traded through the Murray Darling Basin Council [27], but the scientific basis of the regulation has rarely been mentioned. The TWV of the water-rights transaction between Dongyang city and Yiwu city in Zhejiang province is 49.99 million m^3^ per year, and the transaction term is permanent. As time goes by, there has also been a lack of rigorous demonstration as to whether the annual water-saving potential of Hengjin Irrigation District in Dongyang city can still provide the TWV of 49.99 million m^3^. In recent years, with the development of water-rights trading, many scholars begin to put forward the restrictive factors of the TWV from the perspective of the negative effects of water-rights trading. The transfer from agricultural irrigation water to industrial water will change river runoff and exacerbate the dry seasons [28,29]. Therefore, the states that allow water-rights trading in the United States require water administration departments to conduct environmental impact assessments before trading, to determine the reasonable TWV to reduce the negative impacts. Dellapenna [30] believed that water-rights trading may have an impact on the ecological environment, economic development, and the lives of residents in areas where the water rights were transferred. Georgia in the United States stipulates that the TWV in the cross-basin water-rights trading must be the remaining amount of water after meeting the water demand of the basin. Chinese scholars have carried out research on TWV from qualitative and quantitative perspectives. Zhang et al. [31] theoretically defined the total economic water consumption in a dry year as the TWV in a city, and constructed the assumption of the urban water-rights trading mechanism in the arid region of northwest China. Dou et al. [32] took the water-use efficiency red line as the hard constraint and theoretically proposed the working process of water-rights trading, including water-plan formulation, water-saving potential analysis, calculation of water conversion coefficient, calculation of the TWV, and optimization of trading scheme. Based on the Most Stringent Water Resources Management System, Li et al. [33] proposed that calculation of the amount of water rights allowed to be traded and the amount of water taken from actual transactions were the key technical issues of the water-rights trading mechanism, and the water-saving potential of specific regions and industries were the basis of the theory of TWV. Tan [34] considered the difference of water guarantee rate between agriculture and industry, as well as the occupation of industrial water on agricultural water, and calculated the TWV converted from agriculture to industry. The above research has promoted the theory of TWV and water-rights trading.

However, there are still some problems to be solved: (1) The TWV in water-rights transactions is mostly theoretical research with few calculation methods; (2) In the study of water-rights trading, regional water resources are mainly concerned, while industries are the water users. In the practice of water-rights trading, industries are often taken as the basic trading units, and research on the tradable water volumes of industry (ITWV) is lacking. (3) With the increase of water shortage and the control of the total amount of water used by the state, the problem of water resources in water-rich areas is becoming increasingly serious. Therefore, research on water-rights trading in water-rich areas is very urgent.

According to the theory of property rights, this paper puts forward that the ITWV is the amount of water that can be used to carry out the water-rights transaction after the initial water-rights allocation of agriculture, industry, and resident life (including urban resident life and rural resident life) meeting the water demand. Therefore, the ITWV is the difference between the initial water-rights allocation of industry and the water demand of industry. The ITWV affects the preliminary judgment of water-rights trading subjects. In this paper, we applied the calculation method of ITWV to Changsha city in water-rich regions of China, and predicted the ITWV in agriculture, industry, and resident life in different runoff frequencies in 2030, and analyzed the identities of the three industries in water-rights transaction. The paper enriches the research on water-rights transaction in water-rich regions of China.

**Figure 1:**
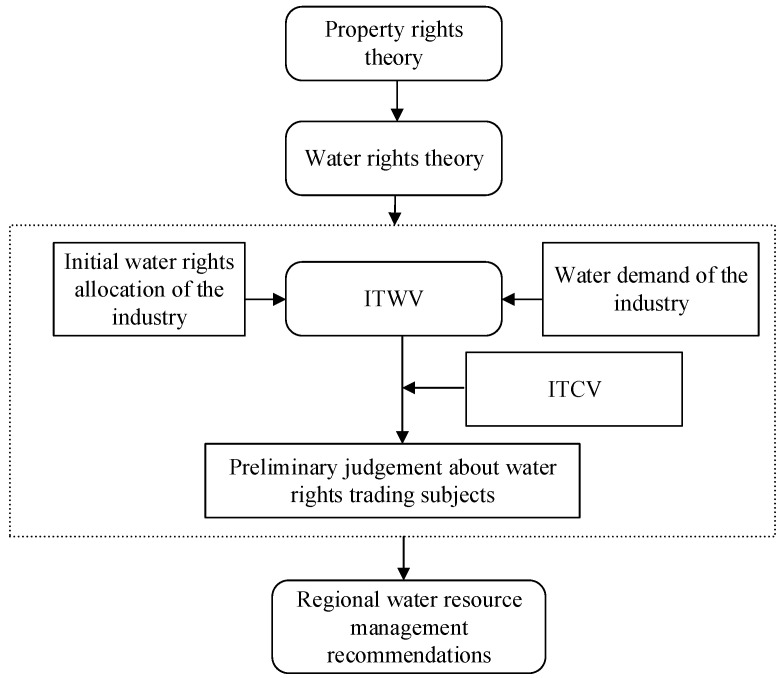
The schematic diagram of the research structure. (ITWV is short for tradable water volumes of industry. ITCV is short for tradable control volumes of industry.).

The rest of this study is organized as follows: In Section 2, the theories of TWV and ITWV are analyzed. In Section 3, the methods are introduced. In Section 4, the study area and the data sources are introduced. In Section 5, the results and discussions are introduced. In Section 6, the study is concluded. An outline is shown in Figure 1.

## 2. The Theories of the TWV and ITWV

### 2.1. Analysis of the TWV Based on Property Rights Theory

From the perspective of new institutional economics, the property rights system is a competition rule established to solve the conflict of competing for scarce resources in human society [35]. With the rapid development of economy and society, the scarcity of water resources is becoming increasingly obvious, which has become a typical economic commodity and even an important factor restricting social development [36]. To strengthen the protection of water resources and other natural resources, China promulgated the Guiding Opinions on Comprehensively Promoting the Reform of the Property rights System of Natural Resources Assets in 2019, which points out that the property rights system is the cornerstone of the socialist market economy. The water-rights system is the concrete embodiment of the property rights system in water-resource use [37]. Therefore, the water-rights system is very important for the allocation efficiency of water resources [38]. The essence of water-rights transaction is the transfer of water-resource use rights, which is an important and basic link in the effective allocation of water resources. The premise of transaction is that water rights have certain exclusivity, and the initial water-rights ownership is clear.

As a special natural resource, water resources play an important role in ensuring economic development and social stability [39,40]. Therefore, water-rights transactions cannot be completely equal to general trading activities. The key point is that the amount of water that the agents participate in a water-rights transaction, i.e., the TWV, is on the premise of meeting the water demand of their own production, life, and the ecology. According to the theory of property rights, the boundary of water-rights transaction is the initial water rights, namely TWV cannot exceed the initial water rights allocated by the state to the subjects. To solve the contradiction between water-resource shortage and social development, and realize the scientific and reasonable allocation of water resources among administrative regions in the basin, it is necessary to carry out the initial water-rights allocation, which is a compulsory regulation of the law or the government [41]. In 2007, China’s Ministry of Water Resources promulgated the Interim Measures for Water Allocation. Since then, different watersheds have advanced their own water allocation, which lays the foundation for defining the initial water rights among the regions. Based on the data from the website of China Water Rights Exchange, most of the water-rights trading practices are between industries, and industries are important participants. Consequently, this paper takes the ITWV as the research perspective. ITWV refers to the water volumes after meeting the water demand of industry within the scope of the initial water rights of industry.

It is worth noting that the notions of the TWV and water-rights trading volumes defined in this paper are different. For the transferor, the TWV is the surplus water after the initial water rights meeting its own water demand; for the transferee, the TWV is the difference between the initial water rights and its own water demand. The transaction volumes of water rights are the transaction objects in the water-rights trading agreement. They are the actual trading volumes considering the transferor and the transferee at the same time. In addition, the concept of the TWV and the amount of water shortage are also distinct. The TWV is proposed from the perspective of property rights, which is within the scope of initial water rights, while the water-shortage volumes are proposed from the perspective of water supply and demand, which is the difference between regional resource supply and demand. The economic development level and water-resource supply capacity of regions differ. In some places, the economic development is fast and the water-resource demand is large, but the water-resource supply cannot meet the demand. The government meets its water-resource demand through administrative water transfer and other ways, and the amount of water demand is determined as the initial water-rights quantities. Thus, the initial water-rights volumes of the region are greater than its water-resource supply amount, which shows that there is not always the same data between the TWV and water-shortage quantities.

Most scholars have carried out extensive research about initial water-rights allocation on river basins or regions, and put forward quantitative methods based on multi-objective decision-making, multi-objective coupling and multi-agent participation [42,43]. The proportion method has advantages in water-resource prediction. It is based on the proportional relationship of historical data to predict the future situations of water resources [44]. In terms of initial water-rights allocation, the proportion method not only considers the historical water allocation scheme, but also takes the regional and industrial water demand into account, which can give a more scientific initial water-rights allocation plan according to the changing situation. Based on the research of initial water rights of industry, this paper used the proportion method to calculate the regional initial water-rights volumes according to the discharged water volumes of basin water distribution control sections, and determined industrial initial water-rights amount according to the historical water-consumption proportion of agriculture, industry, and resident life. There are mature theories and prediction methods about water demand prediction, such as artificial neural network and grey prediction model [45]. Grey system theory is a theoretical science which takes the uncertainty system of “little data and poor information” as the research objects [46], and will achieve good prediction effects in water demand prediction. This paper selected grey model (1,1) (GM (1,1)) to forecast the future water demand according to the historical water consumption of agriculture, industry, and resident life. Due to the water of ecological environment plays a key role in the protection of environment, and urban public water includes more water departments, it is difficult to subdivide in statistics. Therefore, this paper assumed that eco-environmental water and urban public water do not participate in water-rights trading.

### 2.2. Preliminary Judgment of Water-Rights Trading Subjects Based on ITWV and ITCV

The TWV is the difference between the initial water-rights allocation and water demand. In China, the initial water rights are mostly determined by the government according to the regional water-resource situation, under the total water-use control, and considering the historical water allocation scheme. However, the development of regions or industries is different, and the demand for water resources is also dissimilar. Therefore, in industries the value of ITWV can be divided into positive and negative. If the ITWV is a positive number, which indicates that industry has a certain amount of surplus water after ensuring its own water use. Therefore, it can be considered to be the “quasi-transferor” of water-rights transaction. If the ITWV is a negative number, which indicates that the initial water-rights volumes of industry can no longer meet its own water demand. It needs to adopt water-saving technology or purchase water rights to meet its own needs, which is the “quasi-transferee” of water-rights trading.

**Figure 2:**
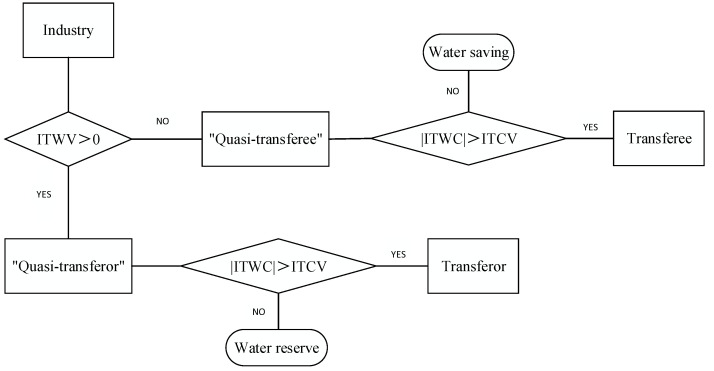
The schematic diagram of preliminary judgment of water-rights transaction subjects. (ITWV is short for tradable water volumes of industry. ITCV is short for tradable control volumes of industry.).

However, it is lack of scientific basis to judge the subjects of water-rights transaction only considering the positive or negative situation of the ITWV. It is necessary to think the absolute value of ITWV. Water-rights trading is an economic activity, it is important to consider the needs of the main bodies of the transaction and economic benefits [47]. From the perspective of “quasi-transferor”, when the ITWV is small, the water-rights transaction cannot meet the water-resource demand of the transferee, and the transaction cannot be carried out due to the lack of trading objects. At this situation, the surplus water can be reserved for development or to cope with drought and other conditions. From the perspective of “quasi-transferee”, in water-rights transaction, the transferee not only needs to pay for water-rights transfer costs, but also invests in water diversion pipelines and water storage projects. When the ITWV is small, the transaction income may be less than the cost, and there is lack of transaction power for the “quasi-transferee”. It could be to construct water-saving projects to solve the problems of insufficient water resources for the “quasi-transferee”. The paper put forward the concept of the tradable control volumes of industry (ITCV). When the absolute value of ITWC is less than the ITCV, the “quasi-transferor” or “quasi-transferee” will not participate in the water-rights transaction, and the management mode of surplus or insufficient water is determined by themselves; when the absolute value of ITWC is greater than the ITCV, the “quasi-transferor” or “quasi-transferee” can participate in the trading. Considering the government’s reserved water quantity and the water-saving potential of industry, the ITCV is taken as 5% of the initial water rights of industry [48,49]. In addition to the ITWV, the determination of water-rights transaction subjects also needs to consider the regional water-use efficiency [50], transaction willingness, and other factors. However, whether there are water volumes available for trading is the prerequisite for the determination of water-rights transaction subjects, so the ITWV is the preliminary basis for the determination of water-rights transaction subjects. The preliminary judgment of water-rights transaction subjects is as shown in Figure 2.

The ITWV not only reflects the trade demand of industry, but also shows the water-use status of industry. According to the Water Law of the People’s Republic of China (revised in 2016), the development and use of water resources should first meet the domestic water needs of urban and rural residents, and consider the demands of agriculture, industry, ecological environment, and shipping, which fully shows the priority of domestic water in water security. Domestic water is the foundation of human survival and development, and the basic guarantee of water human rights. Water human rights is the right of the international communities, countries, enterprises, and individuals to actively protect everyone’s right to enjoy safe, clean, accessible, acceptable and affordable water resources without discrimination [51]. It is one of the human rights. In 2010, the United Nations General Assembly adopted a resolution to raise water human rights to the level of international law, further clarifying the international status of water human rights [52]. Thielbörger analyzed the guarantee measures of each country for water human rights, including making clear daily water quantity, water quality, etc. [53]. Chávarro illustrated that the human rights to water is the premise of protecting the rights to life [54]. Hence, in the transaction of water rights, we should pay attention to the ITWV for resident life, and give priority to the satisfaction of domestic water, to ensure the fairness of water resources.

## 3. Methods

To calculate the ITWV, it is necessary to make clear the regional initial water rights, initial water-rights allocation of industry and water demand of industry. In this paper, we used the proportion method to calculate the regional initial water-rights quantities and initial water-rights allocation of industry under different runoff frequencies, and the GM (1,1) to predict the water demand of industry in the future.

### 3.1. Calculation of the ITWC

ITWC is the difference between the initial water-rights volumes and the water demand of industry, as shown in Equation (1):

(1) $$w_{it}=w_{ia}-w_{id}$$

where *w_it_* represents the ITWC of industry *i* in region (m^3^), *w_ia_* represents the initial water-rights volumes of industry *i* in region (m^3^), *w_id_* represents the water demand of industry *i* in region (m^3^).

### 3.2. Regional Initial Water-Rights Volumes and Industrial Initial Water-Rights Volumes under Different Runoff Frequencies

In this paper, we used the regional initial water-rights allocation proportion under the multi-year average runoff frequency and the discharge volumes of the basin water allocation control section to determine regional initial water rights under different runoff frequencies, as shown in Equation (2):

(2) $$W_{a}^{\eta }=Q^{\eta }\times \varpi$$

where $W_{a}^{\eta }$ represents the regional initial water-rights quantities under the frequency of *η*, (m^3^), *Q^η^* represents the discharged water volumes of the basin water allocation control section under frequency of *η*, (m^3^), *ϖ* represents the regional initial water-rights allocation proportion under the multi-year average runoff frequency.

The initial allocation of water rights should meet the water demand of industry. Therefore, the initial water rights of industry are allocated according to the proportion of industry’s historical water consumption in the total regional water consumption, as shown in Equation (3):

(3) $$w_{ia}=W_{a}^{\eta }\times \nu_{i}=W_{a}^{\eta }\times \frac{\sum_{t=1}^{m}q_{it}}{\sum_{t=1}^{m}Q_{t}}$$

where *q_it_* represents the water consumption of industry *i* in region at the *t*th year (m^3^), *Q_t_* represents the total regional water consumption at the *t*th year (m^3^); *v_i_* represents the initial water-rights allocation proportion of industry *i*. 

### 3.3. Forecast of Industrial Water Demand 

In this paper, we used GM (1,1) [46] to predict the *w_id_*, namely agricultural water demand, industrial water demand, and domestic water demand.

It is known that the original sequence is, *X*^(0)^ = (*x*^(0)^(1), *x*^(0)^, …, *x*^(0)^(*n*)) where *x*^(0)^(*k*) ≥ 0. (*k* = 1, 2, 3, …, *n*) refers to the water consumption of industry over the years, the grade ratio of the sequence should be within the allowable coverage range $\left(e^{-\frac{2}{n+1}},e^{\frac{2}{n+1}}\right)$ To weaken the randomness and volatility of the original sequence, first-order accumulated generating operation sequence is obtained as shown in Equation (4):

(4) $$X^{\left(1\right)}=\left(x^{\left(1\right)}\left(1\right),x^{\left(1\right)}\left(2\right),\cdot \cdot \cdot ,x^{\left(1\right)}\left(n\right)\right)$$

We used the first order univariate differential equation to obtain whitening differential equate of GM (1,1), as shown in Equation (5):

(5) $$\frac{dx^{\left(1\right)}}{dt}+ax^{\left(1\right)}=\mu$$

We obtained the parameters by using least square method, as shown in Equation (6):

(6) $${\left(a,\mu \right)}^{T}={\left(B^{T}B\right)}^{-1}B^{T}Y_{n}$$

where:

(7) $$B=\left[\begin{array}{ccc} -\frac{1}{2}\left(x^{\left(1\right)}\left(1\right)+x^{\left(1\right)}\left(2\right)\right) & & 1\\ -\frac{1}{2}\left(x^{\left(1\right)}\left(2\right)+x^{\left(1\right)}\left(3\right)\right) & & 1\\ \ldots & & \ldots \\ -\frac{1}{2}\left(x^{\left(1\right)}\left(n-1\right)+x^{\left(1\right)}\left(n\right)\right) & & 1 \end{array}\right]$$

(8) $$Y_{n}=\left[\begin{array}{c} x^{\left(0\right)}\left(2\right)\\ x^{\left(0\right)}\left(3\right)\\ \ldots \\ x^{\left(0\right)}\left(n\right) \end{array}\right]$$

The time response formula was as shown in Equation (9):

(9) $$\{\begin{array}{c} {\hat{x}}^{\left(1\right)}\left(k+1\right)=\left(x^{\left(0\right)}\left(1\right)-\frac{\mu }{a}\right)e^{-ak}+\frac{\mu }{a}\\ {\hat{x}}^{0}\left(k+1\right)={\hat{x}}^{\left(1\right)}\left(k+1\right)-{\hat{x}}^{\left(1\right)}\left(k\right) \end{array}\;(k=1,2,3,\ldots ,n)$$

It is necessary to test the accuracy of the model before forecasting. In this paper, we used relative error, absolute correlation degree, and mean square error ratio to test the accuracy of the model.

(1) Relative error Δ(*k*):

(10) $$\Delta \left(k\right)=\left|\frac{\varepsilon \left(k\right)}{x^{\left(0\right)}\left(k\right)}\right|\;(k=1,2,3,\ldots ,n)$$

where *ε*(*k*) represents the model residual. For a given model relative error $\bar{\Delta }$, if Δ(*k*) > $\bar{\Delta }$, the model is called relative error qualified model.

(2) Absolute correlation degree *ς*:

(11) $$\varsigma =\frac{1+\left|s\right|+\left|\hat{s}\right|}{1+\left|s\right|+\left|\hat{s}\right|+\left|\hat{s}-s\right|}$$

where

(12) $$\left|s\right|=\left|\sum_{k=2}^{n-1}\left(x\left(k\right)-x\left(1\right)\right)+\frac{1}{2}\left(x\left(n\right)-x\left(1\right)\right)\right|$$

(13) $$\left|\hat{s}\right|=\left|\sum_{k=2}^{n-1}\left(\hat{x}\left(k\right)-\hat{x}\left(1\right)\right)+\frac{1}{2}\left(\hat{x}\left(n\right)-\hat{x}\left(1\right)\right)\right|$$

For a given absolute correlation degree *ς*_0_ of a model, if ς > *ς*_0_, the model is called an absolute correlation qualified model.

(3) Mean square error ratio *C*:

(14) $$C=\sqrt{\frac{S_{2}^{2}}{S_{1}^{2}}}$$

where $S_{1}^{2}$ represents the variance of the original sequence, $S_{2}^{2}$ represents the variance of the residual sequence. For mean square error ratio *C*_0_ of a given model if *C* > *C*_0_, the model is called a qualified model of mean square deviation ratio. Table 1 is the reference of model accuracy inspection grades. From Class I to Class IV, the accuracy of the model is getting lower and lower.

## 4. Study Area and Data

### 4.1. Study Area

**Figure 3:**
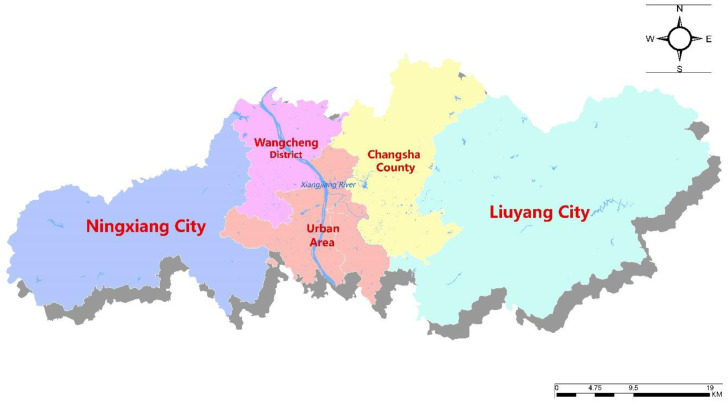
The administrative division map of Changsha city.

Changsha city is in the eastern part of Hunan Province and the lower reaches of the Yangtze River, it is an important city in Hunan Province. It is between 111°53′–114°15′ E and 27°51′–28°41′ N. In 2019, Changsha city had a total area of 11819 square kilometers, a total resident population of 8.3945 million, and the urbanization rate was 79.56%. Changsha city is in the subtropical monsoon climate zone, with the annual average temperature between 16.8 °C and 17.3 °C, and the annual average rainfall is 1483.36 mm. The precipitation is mainly concentrated from April to June, accounting for 51% of the whole year, it has the characteristics of spatial and temporal distribution differences [55]. The annual average surface runoff is 8.265 billion m^3^ and the annual average evaporation is 1206.09 mm, which is a typical water-rich area in China. Changsha city is composed of urban area, Wangcheng district, Changsha county, Liuyang city, and Ningxiang city. The main rivers are Xiangjiang River and its primary tributaries: Liuyang River, Laodao River, and Weishui River, the administrative division map is as shown in Figure 3.

In recent years, the economic development of Changsha city is rapid. In 2019, the gross domestic product (GDP) of Changsha city was CNY 1157.422 billion, ranking the 15th in China and 1st in Hunan Province. The added value of the primary industry was CNY 35.969 billion, an increase of 3.2%; the added value of the secondary industry was CNY 443.932 billion, an increase of 8%; the added value of the tertiary industry was CNY 677.521 billion, an increase of 8.4% [56]. Although Changsha city is in abundant water areas, the total amount of water resources is rich, but with the rapid expansion of urban population, the acceleration of industrialization, and urbanization process, the demand for water is growing vigorously, and the contradiction between the shortage of water resources and the rapid social development is increasingly obvious. In 2019, the total water resources of Changsha city were 10.12 billion m^3^, ranking the 10th among the 14 cities (prefectures) in the province, while the water consumption is 3.585 billion m^3^, ranking the 3rd in the province. The development and use rate of water resources in the city has reached 37.3%, while it is generally believed that the rate should not exceed 40%, which indicates that the development and use of water resources in Xiangjiang River Basin is at a high level. The effective use coefficient of farmland irrigation water was 0.5451, while that of foreign advanced water-saving irrigation countries had reached 0.7–0.8. In 2018, the repetition rate of industrial water in Changsha city was 65.63%, far lower than the national average of 90.13%, and the leakage rate of urban pipe network was 14.63% [57], the advanced level of foreign countries was about 10%. In 2012, China implemented the Most Stringent Water Resources Management System, which developed higher requirements for regional total water consumption and water-use efficiency. To solve the problem of water shortage in urban development in the new period, Changsha city should adhere to the policy of “water-saving priority, spatial balance, systematic governance and two hands working together”, introduce market mechanisms and give full play to the role of water-rights trading.

At the beginning of 2019, the Water Resources Departments of Hunan Province printed and issued the Water Allocation Plan of Main River Basins. In combination with the water-resource zoning situations designated by the state, Hunan Province allocated the total water-consumption control index to the basins, and proposed the average water allocation share of each prefecture level administrative region in basin, and completed the regional initial water-rights allocation, which laid the foundation for the development of water-rights trading. On 31 July 2019, Tongrenqiao Management Office of Changsha County in Hunan Province completed the repurchase of agricultural irrigation water rights in Tongrenqiao Irrigation Area in 2018 through the national water-rights trading platform, which provided reference for the exploration and practice of water-rights trading in the province. Water-rights trading will be a beneficial attempt to optimize the allocation of water resources in Changsha city and even Hunan Province in the future.

### 4.2. Data Sources

The data of discharge water quantities of Xiangjiang River Section-Xiangtan city under different runoff frequencies is from the document of Water Resources Department of Hunan Province, which is as shown in Table 2. According to the document, in 2030, the initial water-rights volumes of Changsha city will be 4100 million m^3^ under the multi-year average runoff frequency. 

The data of agricultural water consumption, industrial water consumption, domestic water consumption and total water consumption of Changsha city are from the Water Resources Bulletin of Changsha City from 2010 to 2019, as shown in Table 3. According to Table 3, the proportions of agricultural water consumption, industrial water consumption and domestic water consumption in the total water consumption from 2010 to 2019 are as follows: *v*_1_ = 43.91%, *v*_2_ = 35.54%, *v*_3_ = 10.61% (1, 2 and 3 respectively represents agriculture, industry, and resident life.)

## 5. Results and Discussions

### 5.1. Regional Initial Water-Rights Volumes under Different Runoff Frequencies in 2030

According to the proportion of initial water rights of Changsha city to the discharged water volumes of Xiangjiang River Section–Xiangtan city under the multi-year average runoff frequency, we calculated the initial water-rights allocation amount of Changsha city in 2030 under different runoff frequencies. The results are as shown in Table 4.

### 5.2. The ITWC of Agriculture under Different Runoff Frequencies in Changsha City in 2030 

#### 5.2.1. Agricultural Initial Water-Rights Volumes under Different Runoff Frequencies in Changsha City in 2030

According to the proportion of agricultural water consumption in the total water consumption from 2010 to 2019, the initial water rights of agriculture in Changsha city in 2030 under different runoff frequencies are shown in Table 5.

#### 5.2.2. Forecast of Agricultural Water Demand in Changsha City in 2030

According to the grey prediction theory, the GM (1,1) of agricultural water demand in Changsha city is as shown in Equation (15):

(15) $${\hat{x}}_{agr}^{\left(1\right)}\left(k\right)=-757.2723e^{-0.0238k}+757.0336\;k=1,\;2,\;3,\;\ldots ,\;n$$

The model fitting results are shown in Table 6.

The average variance ratio *C* = 0.2835, the correlation degree *ς* = 0.9878, the relative error is low. Therefore, Changsha agricultural water demand prediction model meets the accuracy requirements. The development coefficient −*a* is less than 0.3, and the prediction model can be used for medium and long-term prediction [46]. According to Equation (15), the agricultural water demand of Changsha city in 2030 is 1133.14 million m^3^.

#### 5.2.3. Calculation of Agricultural ITWV under Different Runoff Frequencies in Changsha City in 2030

According to the calculation results of agricultural initial water-rights allocation, agricultural water demand and Equation (1), it can be obtained the ITWV and the ITCV of agriculture in Changsha city in 2030 under different runoff frequencies are as shown in Table 7.

#### 5.2.4. Analysis of Agricultural ITWV under Different Runoff Frequencies in Changsha City in 2030

1. In 2030, under the frequency of 50% and 75%, the ITWV of agriculture in Changsha city is 611.48 million m^3^ and 267.47 million m^3^ respectively, which are greater than the agricultural ITCV of 87.23 million m^3^ and 70.03 million m^3^. It shows that the agricultural initial water-rights volumes can meet water demand, but also have more residual water. It could be as the transferor of water-rights transaction to participate in the water-rights transaction and transform the resource benefits into economic benefits.
2. In 2030, under the frequency of 90%, the ITWV of agriculture in Changsha city is −38.22 million m^3^, which indicates that the initial agricultural water rights can no longer meet its water demand, and agricultural production may be affected, which is not conducive to ensuring regional food production and social stability. However, due to the absolute value of agricultural ITWV is less than the ITCV of 54.75 million m^3^, it shows that the water-saving potential of agriculture can make up for this part of water shortage. Agricultural departments could take measures to adjust agricultural planting structure, improve the effective use coefficient of farmland irrigation water, and reduce the irrigation quota to improve the water-saving potential and meet the agricultural water demand.

### 5.3. The ITWC of Industry under Different Runoff Frequencies in Changsha City in 2030 

#### 5.3.1. Industrial Initial Water-Rights Volumes under Different Runoff Frequencies in Changsha City in 2030

According to the proportion of industrial water consumption in the total water consumption from 2010 to 2019, the initial water rights of industry in Changsha city in 2030 under different runoff frequencies are shown in Table 8.

#### 5.3.2. Forecast of Industrial Water Demand in Changsha City in 2030

According to the grey prediction theory, the GM (1,1) of industrial water demand in Changsha city is as shown in Equation (16):

(16) $${\hat{x}}_{ind}^{\left(1\right)}\left(k\right)=-1042.933e^{-0.0167k}+1056.1481\;k=1,\;2,\;3,\;\ldots ,\;n$$

The model fitting results are shown in Table 9.

The average variance ratio *C* = 0.3059, the correlation degree *ς* = 0.9118, the relative error is low. Therefore, Changsha industrial water-consumption prediction model meets accuracy requirements. The development coefficient −*a* is less than 0.3, and the prediction model can be used for medium and long-term prediction [46]. According to Equation (16), industrial water demand of Changsha city in 2030 is 1082.39 million m^3^.

#### 5.3.3. Calculation of Industrial ITWV under Different Runoff Frequencies in Changsha City in 2030

According to the calculation results of industrial initial water-rights allocation, industrial water demand and Equation (1), it can be obtained the ITWV and the ITCV of industry in Changsha city in 2030 under different runoff frequencies are as shown in Table 10.

#### 5.3.4. Analysis of Industrial ITWV under Different Runoff Frequencies in Changsha City in 2030

1. In 2030, under the frequency of 50%, industrial ITWV of Changsha city is 329.69 million m^3^, which is greater than industrial ITCV of 70.6 million m^3^. It shows that industrial initial water-rights volumes can meet its water demand, and there is a relatively surplus water, which can be adjusted to other industries or regions as the transferor of water-rights transaction.
2. In 2030, under frequency of 75%, industrial ITWV of Changsha city is 51.25 million m^3^, but less than industrial ITCV of 56.68 million m^3^. This indicates that although the initial industrial water-rights volumes can meet its water demand, the residual water is not enough to sell to the transferee. Therefore, this part of the water can be reserved and used to support industrial development or to deal with pollution and other unconventional use of water.
3. In 2030, under frequency of 90%, industrial ITWV of Changsha city is −196.18 million m^3^, and its absolute value is greater than the ITCV of 44.31 million m^3^, which represents that industrial initial water-rights quantities cannot meet its water demand, and industrial water shortage easily leads to the decline of output value. Therefore, industry can be considered to be the transferee to solve the problem of water shortage through water-rights transaction.

### 5.4. The ITWC of Resident Life under Different Runoff Frequencies in Changsha City in 2030 

#### 5.4.1. Domestic Initial Water-Rights Volumes under Different Runoff Frequencies in Changsha City in 2030 

According to the proportion of domestic water consumption in the total water consumption from 2010 to 2019, the initial water rights of resident life in Changsha city in 2030 under different runoff frequencies are as shown in Table 11.

#### 5.4.2. Forecast of Domestic Water Demand in Changsha City in 2030

According to the grey prediction theory, the GM (1,1) of domestic water demand in Changsha city is as shown in Equation (17):

(17) $${\hat{x}}_{life}^{\left(1\right)}\left(k\right)=267.2234e^{0.0102k}-263.0217\;k=1,\;2,\;3,\;\ldots ,\;n$$

The model fitting results are shown in Table 12.

The average variance ratio *C* = 0.2253, the correlation degree *ς* = 0.9909, the relative error is low. Therefore, Changsha domestic water-consumption prediction model meets the accuracy requirements. The development coefficient −*a* is less than 0.3, and the prediction model can be used for medium and long-term prediction [46]. According to Equation (17), the domestic water demand of Changsha city in 2030 is 482.64 million m^3^.

#### 5.4.3. Calculation of Domestic ITWV under Different Runoff Frequencies in Changsha City in 2030

According to the calculation results of domestic initial water-rights allocation, domestic water demand and Equation (1), it can be obtained the ITWV and the ITCV of resident life in Changsha city in 2030 under different runoff frequencies are as shown in Table 13.

#### 5.4.4. Analysis of Domestic ITWV under Different Runoff Frequencies in Changsha City in 2030

In 2030, under frequency of 50%, 75% and 90%, the domestic ITWV in Changsha city is −61.24 million m^3^, −144.33 million m^3^ and −218.17 million m^3^ respectively, and the absolute values are greater than the domestic ITCV of 21.07 million m^3^, 16.92 million m^3^ and 13.22 million m^3^. It shows that the initial water rights of resident life cannot meet the needs of people, and the water shortage has affected people’s normal life. The government could consider solving the problem of insufficient domestic water through water-rights trading. Domestic water is the foundation of human survival and development, and the basic guarantee of water human rights. Water human rights is one of the basic contents of human rights and must be guaranteed in priority. Therefore, Changsha city needs to pay attention to the problem of insufficient domestic water and take measures to actively solve it.

### 5.5. Analysis of Water-Resource Management in Changsha City in 2030 under Different Runoff Frequencies

**Figure 4:**
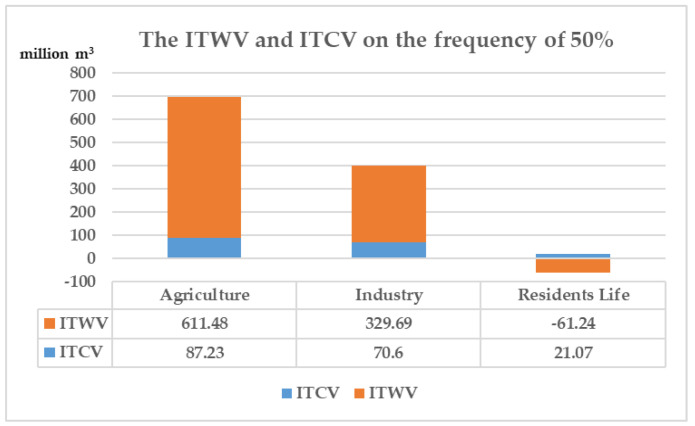
The ITWV and ITCV of industry on the frequency of 50%. (ITWV is short for tradable water volumes of industry. ITCV is short for tradable control volumes of industry.).

In 2030, under the frequency of 50%, the ITWV of agriculture and industry in Changsha city are greater than their respective ITCV, so they could be considered to be the transferors to participate in water-rights trading. The domestic water is in shortage, the ITWV is within the range of ITCV, hence water-saving measures can be taken to solve the problem of water shortage. The ITWV of agriculture or industry can supplement the shortage of domestic water, and the optimal allocation of water resources can be realized by adjusting the surplus and shortage among industries. The ITWV and ITCV of industry on the frequency of 50% are as shown in Figure 4.

**Figure 5:**
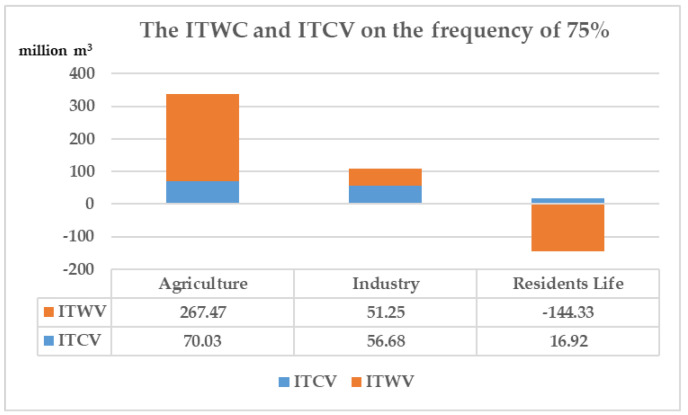
The ITWV and ITCV of industry on the frequency of 75%. (ITWV is short for tradable water volumes of industry. ITCV is short for tradable control volumes of industry.).

In 2030, under the frequency of 75%, the ITWV of agricultural in Changsha city is greater than the ITCV, so it can be considered to be the transferor to participate in water-rights trading. Industrial ITWV is within the range of the ITCV, consequently the surplus water is considered to be stored for self-use. The domestic water is in a state of shortage, but since its ITWV has exceeded the scope of the ITCV, agriculture can be considered to supplement the ITWV of resident life first. The ITWV and ITCV of industry on the frequency of 75% are as shown in Figure 5.

**Figure 6:**
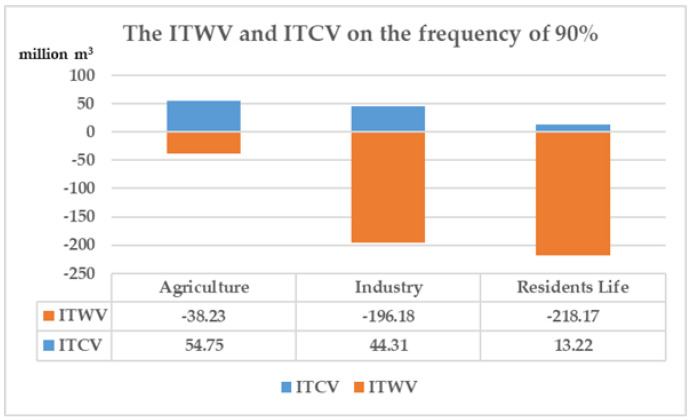
The ITWV and ITCV of industry on the frequency of 90%. (ITWV is short for tradable water volumes of industry. ITCV is short for tradable control volumes of industry.).

At the frequency of 90%, agricultural ITWV, industrial ITWV, and domestic ITWV are all in state of shortage. Although agricultural ITWV can be self-supplemented by water-saving measures, the ITWV of industry and resident life are beyond the ITCV separately. In this situation, Changsha city needs to consider the adoption of cross-region water-rights trading to solve the problem of water shortage. The ITWV and ITCV of industry on the frequency of 90% are as shown in Figure 6.

From the above analyses, water-resource management should combine water-saving with water-rights trading in Changsha city. The contradiction between water shortage and urban development is becoming increasingly acute, water-saving is one of the important ways to realize China’s sustainable development. As the capital city of Hunan Province, Changsha city should take the construction of economical society as one of the goals of urban development. At the same time, the city should actively participate in the market mechanisms, improve the secondary allocation of water resources by using water-rights trading, and take water-rights trading as an auxiliary measure to deal with the shortage of water resources.

### 5.6. Comparison with Other Analyses about the ITWV

Compared with the previous qualitative analysis about the TWV [32], the paper proposed the quantitative method to calculation the ITWV. The method is based on the theory of property rights, and its premise is to make clear the initial water rights of the region or industry, which reflects the essence of water-rights transaction as an economic activity. However, most of the previous studies had not made clear the boundary of TWV, replacing initial water rights with water consumption [31], consequently the results were often lack of science. Therefore, the method proposed in this paper is more scientific and practical. It lays a foundation for scientific determination of ITWV and promotes research of water-rights trading.

## 6. Conclusions 

From the perspective of the ITWV, this paper provides a theoretical basis for the comprehensive management of water resources in water-rich areas and the motivation of industry to develop water-rights trading: (1) Based on the property rights theory, we proposed that the ITWV is the amount of water after meeting the water demand of industry within the scope of industrial initial water-rights volumes, and it is the difference between the initial water rights and the water demand of industry; (2) We proposed that the primary determination of water-rights trading subjects is realized by the relationship between the ITWV and ITCV. When the absolute value of ITWV is less than the ITCV, industry is not recommended to carry out water-rights trading; when the absolute value of ITWV is greater than the ITCV, industry is encouraged to participate in water-rights trading.

In this paper, we used the proportion method to calculate the initial water rights of industry under different runoff frequencies, and GM (1,1) to predict the water demand of industry, to obtain the ITWV. We applied the method to Changsha city, which is in China’s abundant water areas. The results are as follows:

1. In 2030, under the frequency of 50% and 75%, the ITWV of agricultural in Changsha city is 611.48 million m^3^ and 267.47 million m^3^, which is larger than the ITCV of agriculture, so agriculture can be considered to be the transferor of water-rights transaction; under the frequency of 90%, the ITWV of agriculture in Changsha city is −38.23 million m^3^, and its absolute value is within the range of agricultural ITCV, which indicates that agriculture can take various measures to improve water-saving potential to meet water demand.
2. In 2030, under the frequency of 50%, industrial ITWV of Changsha city is 329.69 million m^3^, which is larger than industrial ITCV, so industry can be considered to be the transferor of water-rights transaction; under the frequency of 75%, industrial ITWV of Changsha city is 51.25 million m^3^, less than the ITCV, which can be considered the water for self-use; under frequency of 90%, industrial ITWV of Changsha city is −196.18 million m^3^, and its absolute value is beyond the range of the ITCV, which indicates that industry can consider as the transferee of water-rights transaction to participate in transaction to meet water demand.
3. In 2030, under the frequency of 50%, 75% and 90%, the domestic ITWV in Changsha city is −61.24 million m^3^, −144.33 million m^3^ and −218.17 million m^3^ respectively, and the absolute values of the ITWV are all greater than the ITCV. Therefore, the domestic initial water rights cannot meet the demand of domestic water. Therefore, the problem of insufficient domestic water can be solved through water-rights trading.
4. Water-resource management in Changsha city should combine water conservation with water-rights trading, take the construction of a conservation-oriented society as one of the objectives of urban development, and use scientific measures to widely promote water conservation. At the same time, the government should make water-rights trading an important way to effectively solve the shortage of water resources, actively participate in the reform of water-rights markets, flexibly use market mechanisms to deal with water shortage, and ensure regional development.

Research of the ITWV affects the improvement of water-rights market. The regional water-resource management department should adopt various technical means to monitor and analyze the change of the ITWV and understand in a timely manner the water-use situation and the demand of water-rights trading. The calculation and analysis of ITWV and ITCV should be important steps before trading, which play a key role in effectively identifying trading subjects, determining the trading volumes, and promoting the progress of water-rights markets.

Research about the ITWV is complex. This paper has some limitations: the transfer of water has a certain impact on the health of the water environment, how to reduce the negative impact of water-rights trading on water environment and scientifically define the appropriate ITWV is worthy of further research; the influencing factors of industry water demand are multifaceted, especially at present, and China vigorously promotes water-saving work, so only using historical water-consumption data to predict water demand may ignore the influence of key factors; the analysis and calculation of the ITCV needs to be further studied. They are the directions of future papers.

## Figures and Tables

**Table 1:** The reference of model accuracy inspection grades.

Accuracy Class	Relative Error Δ(*k*)	Absolute Correlation Degree *ς*	Mean Square Error Ratio *C*
Class I	0.01	0.90	0.35
Class II	0.05	0.80	0.50
Class III	0.10	0.70	0.65
Class IV	0.20	0.60	0.80

**Table 2:** The discharge volumes of Xiangjiang River Section-Xiangtan City under different runoff frequencies.

Runoff Frequency	Discharge Volumes of Xiangtan City (Million m^3^)
Annual average	63,020
50%	61,070
75%	49,028
90%	38,327

**Table 3:** Agricultural water consumption, industrial water consumption, domestic water consumption, and total water consumption in Changsha city from 2010 to 2019.

Year	Agricultural Water Consumption (Million m^3^)	Industrial Water Consumption (Million m^3^)	Domestic Water Consumption (Million m^3^)	Total Water Consumption (Million m^3^)
2010	1776.13	1321.51	420.17	3800.06
2011	1747.92	1343.95	398.52	3765.2
2012	1657	1484.63	371.12	3803.33
2013	1647.09	1407.98	376.8	3830.74
2014	1793.63	1317.43	369.93	3838.48
2015	1704.08	1257.77	383.56	3736.94
2016	1654.34	1235.01	387.39	3663.35
2017	1575.87	1280.18	407.61	3667.8
2018	1415.86	1308.37	412.5	3586.39
2019	1400.95	1295.29	427.18	3584.79

**Table 4:** The initial water rights of Changsha city under different runoff frequencies in 2030.

Runoff Frequency	The Discharged Water Volumes of Xiangjiang River Section-Xiangtan City (Million m^3^)	The Initial Water-Rights of Changsha City (Million m^3^)
Annual average	63,020	4100
50%	61,070	3976
75%	49,028	3192
90%	38,327	2495

**Table 5:** The agricultural initial water rights of Changsha city under different runoff frequencies in 2030.

Runoff Frequency	50%	75%	90%
Agricultural Initial Water-Rights Volumes (million m^3^)	1745	1401	1095

**Table 6:** Fitting results of agricultural water demand prediction model in Changsha city from 2010 to 2019.

Year	True Value of Agricultural Water Consumption (Million m^3^)	Fitting Value of GM (1,1) (Million m^3^)	Residual	Relative Error
2010	1776.13	1776.13	0	0
2011	1747.92	1781.03	−33.11	0.0189
2012	1657	1739.14	−82.14	0.0496
2013	1647.09	1698.24	−51.15	0.0311
2014	1793.63	1558.30	235.33	0.1312
2015	1704.08	1719.29	−15.21	0.0089
2016	1654.34	1581.22	73.12	0.0442
2017	1575.87	1544.02	31.85	0.0202
2018	1415.86	1507.71	−91.85	0.0649
2019	1400.95	1472.25	−71.30	0.0509

**Table 7:** The ITWV and the ITCV of agriculture in Changsha city under different runoff frequencies in 2030.

Index	Runoff Frequency		
	50%	75%	90%
Agricultural ITCV (million m^3^)	87.23	70.03	54.75
Agricultural ITWV (million m^3^)	611.48	267.47	−38.23

**Table 8:** Industrial initial water rights of Changsha city under different runoff frequencies in 2030.

Runoff Frequency	50%	75%	90%
Industrial Initial Water-Rights Volumes (million m^3^)	1412	1134	886

**Table 9:** Fitting results of industrial water demand prediction model in Changsha city from 2010 to 2019.

Year	True Value of Industrial Water Consumption (Million m^3^)	Fitting Value of GM (1,1) (Million m^3^)	Residual	Relative Error
2010	1321.51	1321.51	0	0
2011	1343.95	1398.5	54.55	0.0406
2012	1484.63	1379.74	104.89	0.0707
2013	1407.98	1361.25	46.73	0.0332
2014	1317.43	1342.99	−25.56	0.0194
2015	1257.77	1324.98	−67.21	0.0534
2016	1235.01	1307.22	−72.21	0.0585
2017	1280.18	1289.68	−9.5	0.0074
2018	1308.37	1272.4	35.97	0.0275
2019	1295.29	1255.33	39.96	0.0309

**Table 10:** The ITWV and the ITCV of industry in Changsha city in 2030 under different runoff frequencies.

Index	Runoff Frequency		
	50%	75%	90%
Industrial ITCV (million m^3^)	70.6	56.68	44.31
Industrial ITWV (million m^3^)	329.69	51.25	−196.18

**Table 11:** The domestic initial water-rights volumes under different runoff frequencies in 2030.

Runoff Frequency	50%	75%	90%
Domestic Initial Water-Rights Volumes (million m^3^)	421	338	264

**Table 12:** Fitting results of domestic water demand prediction model in Changsha city from 2010 to 2019.

Year	True Value of Domestic Water Consumption (Million m^3^)	Fitting Value of GM (1,1) (Million m^3^)	Residual	Relative Error
2010	420.17	420.17	0	0
2011	398.52	371.32	27.20	0.0683
2012	371.12	376.49	−5.37	0.0145
2013	376.8	381.71	−4.91	0.0130
2014	369.93	387.02	−17.09	0.0462
2015	383.56	392.40	−8.84	0.0230
2016	387.39	397.85	−10.46	0.0270
2017	407.61	403.38	4.23	0.0104
2018	412.5	4.08.99	3.51	0.0085
2019	427.18	4.14.66	12.52	0.0293

**Table 13:** The domestic ITWV and ITCV in Changsha city in 2030 under different runoff frequencies.

Index	Runoff Frequency		
	50%	75%	90%
Domestic ITCV (million m^3^)	21.07	16.92	13.22
Domestic ITWV (million m^3^)	−61.24	−144.33	−218.17

## Abbreviations

TWC	Tradable water volumes
ITWC	Tradable water volumes of industry
ITCV	Tradable control volumes of industry
GM (1,1)	Grey model (1,1)
